# Isolated right atrial rupture from blunt trauma: a case report with systematic review of a lethal injury

**DOI:** 10.1186/s13019-019-0836-8

**Published:** 2019-02-04

**Authors:** Tareq Maraqa, Mohamed A. T. Mohamed, Kenneth L. Wilson, Vinu Perinjelil, Gul R. Sachwani-Daswani, Leo Mercer

**Affiliations:** 1 Trauma Department, Hurley Medical Center, One Hurley Plaza, Flint, MI 48503 USA; 2 Department of Surgery, Michigan State University College of Human Medicine, Eyde Building, Suite 600, 4660 S. Hagadorn Road, East Lansing, MI 48823 USA; 3 Department of Surgery, University of Chicago, 5841 S. Maryland Avenue, Chicago, IL 60637 USA

**Keywords:** Isolated right atrial rupture, Blunt chest trauma, Cardiac rupture, Blunt cardiac rupture

## Abstract

**Background:**

Isolated right atrial rupture (IRAR) from blunt chest trauma is rare. There are no physical exam findings and non-invasive testing specific to the condition, which result in diagnostic delays and poor outcomes. We present a case of IRAR along with a systematic review of similar cases in the literature.

**Case report:**

A 23-year-old male presented following a motor vehicle accident (MVA). He was bradycardic and hypotensive during transportation; and required intubation. There were contusions along the right chest wall with clear breath sounds, and no jugular venous distension, muffled heart sounds. Hemodynamic status progressively worsened, ultimately leading to his death. However, no external sources of bleeding or evidence of cardiac tamponade was found.

**Methods:**

A search of PubMed, Ovid, and the Cochrane Library using: (Blunt OR Blunt trauma) AND (Laceration OR Rupture OR Tear) AND (Right Atrium OR Right Atrial). Articles were included if they were original articles describing cases of IRAR.

**Results:**

Forty-five reports comprising seventy-five (*n* = 75) cases of IRAR.

**Conclusion:**

IRAR most commonly occurs following MVAs as the result of blunt chest trauma. Rupture occurs at four distinct sites and is most commonly at the right atrial appendage. IRAR is a diagnostic challenge and requires a high index of suspicion, as patients’ hemodynamics can rapidly deteriorate. The presentations vary depending on multiple factors including rupture size, pericardial integrity, and concomitant injuries. Cardiac tamponade may have a protective effect by prompting the search for a bleeding source. A pericardial window can be diagnostic and therapeutic in IRAR. Outcomes are favourable with timely recognition and prompt surgical intervention.

## Introduction

Isolated right atrial rupture (IRAR) is a rare injury that occurs secondary to blunt chest trauma. Blunt cardiac rupture (BCR) of the right atrium (RA) has a reported incidence between 0.2–0.5% and occurs concomitantly with BCR of another cardiac chamber [1–3]. Only 10% of patients survive long enough to make it to a hospital [3]. IRAR has heterogeneous clinical manifestations and non-specific laboratory test findings which can delay time for life-saving interventions [4–6].

Due to the paucity in available literature, our knowledge of the condition is limited however cases of IRAR are more commonly encountered than previously thought. Herein, we present a case of IRAR following blunt chest trauma. We also conducted a systematic review of previously reported cases with the primary objective of highlighting unique features of the condition, including its etiology, anatomy, presentations, and outcomes; as well as previously reported practices in diagnosis and management.

## Case report

A 23-year-old man was flown in to our level I trauma center following an MVA collision about 2–3 h prior. En route, he became bradycardic and required intubation after losing consciousness. On presentation to our hospital, his vitals were as follows: heart rate 116-bpm; blood pressure 252/183-mmHg; respiratory rate of 19; and SpO_2_ of 98% on mechanical ventilation.

On examination, the patient was unconscious with dilated and unreactive eyes/pupils. He had several superficial abrasions and an open left femur fracture. There was a 9 × 7 cm contusion along the right chest wall, but chest was clear to auscultation without muffled heart sounds. Carotid, femoral and distal extremity pulses were all 1+ bilaterally without jugular venous distension. He also had mild abdominal distension, left flank ecchymosis, and absent rectal sphincter tone.

Focused assessment with sonography for trauma (FAST) only showed scant abdominal fluid and was negative for tamponade. Bilateral chest tubes were inserted due to suspicion of hemothorax, but produced minimal drainage. The patient deteriorated rapidly despite further resuscitative efforts, which were eventually discontinued at time of death.

Subsequent autopsy revealed an isolated tear in the right cardiac atrium at the junction of the inferior vena cava (IVC). Other findings included bilateral pulmonary contusions, blood in the pleural and peritoneal cavities, and small lacerations of the spleen and liver.

## Methods

A search of all original studies was conducted using PubMed, Ovid, and the Cochrane Library. The following search was performed in PubMed in December 2016: (Blunt OR Blunt trauma) AND (Laceration OR Rupture OR Tear) AND (Right Atrium OR Right Atrial). Publications were limited to publish date after 1955. Language was limited to English. Search was limited to full text. The search was repeated in Ovid and Cochrane library. Abstracts were reviewed, and those that met the selection criteria specified later were imported into a Microsoft Excel sheet. If the full abstract was unavailable or was ambiguous, the article was complied, obtained and reviewed for inclusion.

**Fig. 1:**
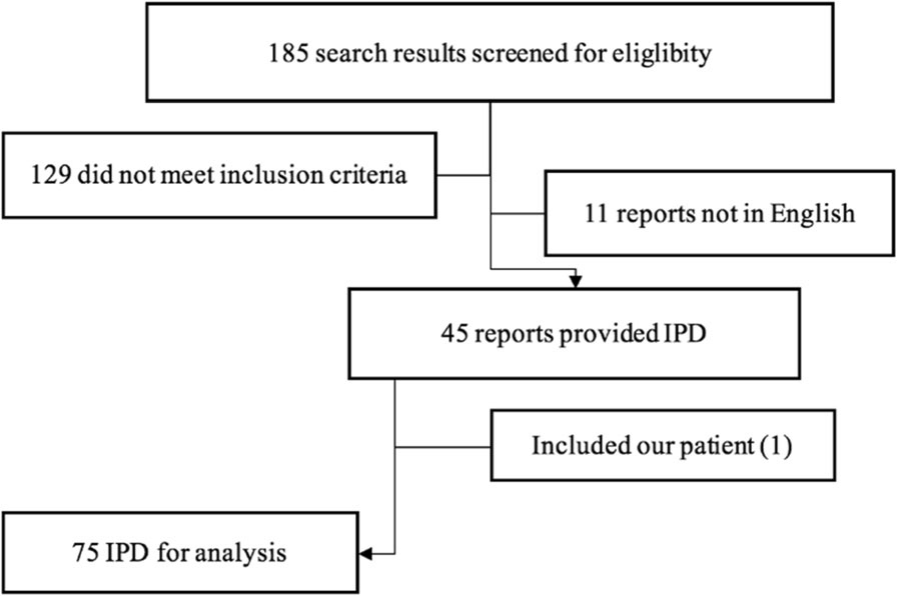
PRISMA IPD flow diagram for methods and patient selection. IPD, Individual Patient Data

Inclusion criteria were (1) original articles describing cases of IRAR due to blunt trauma, (2) basic individual patient data (age, mechanism, clinical presentations, associated injuries, etc.) were presented independently. Institutional studies that reported only incidence were excluded, as well literature reviews. A summary of our methods using the "PRISMA IPD flow diagram for methods and patient selection is illustrated in (Fig. 1).

## Results

A total of 137 titles and abstracts were screened. A total of 47 original articles met the selection criteria. A total of 75 individual patient data was retrieved, including our patient, and compiled for analysis (Table 1) [4–47].

**Table 1:** Results and summary of demographics, sites of rupture, presentations, and outcomes

No.	75
Age, years	26 (0–75)
Male	71% (53)
Female	29% (22)
MVA	92% (69)
Other cause	8% (6)
Site of RA rupture	
Right atrial appendage	35% (26)
RA/SVC junction	17% (13)
RA/IVC junction	21% (16)
Free RA wall	15% (11)
Multiple RA sites	12% (9)
Clinical presentation	
Hypotension	100% (75)
Cardiac tamponade	65% (49)
Lung contusion	25% (19)
Hemothorax	28% (21)
Right-sided	76% (16)
Left-sided	5% (1)
Bilateral	19% (4)
Outcomes	
Mortality	11% (8)
Hospital LOS	10 (0–99)

Median (range); proportion % (n); MVA, motor vehicle accident; RA, right atrium; SVC/IVC, superior/inferior vena cava; LOS, length of stay

The median age was 26-years and affected all ages, from new-borns to elderly (0–75). The vast majority of cases occurred secondary to a MVA (92%). There are four sites in the RA where IRAR occurs and the Right Atrial Appendage (RAA) was the most common site of rupture (35%). All patients presented with hypotension and physical exam findings suggestive of cardiac tamponade were also common (62%).

**Fig. 2:**
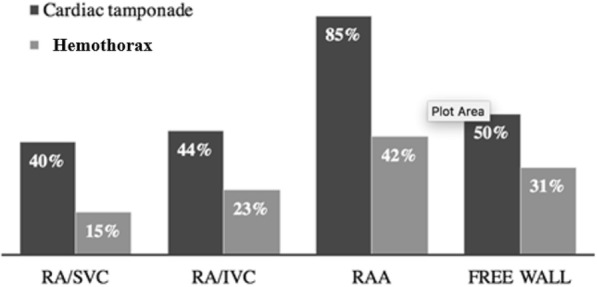
Incidences of cardiac tamponade and hemothorax at different right atrial rupture sites

There was a statistically significant difference between the averages length of stay (LOS) with each rupture sites, specifically the LOS was doubled when IRAR occurred at the free RA wall. We also observed a statistically significant difference between the rupture sites and whether they present with cardiac tamponade. Specifically, 85% of IRAR at the RAA presented with cardiac tamponade, which could suggest that the location of IRAR may predict presentation (Fig. 2). However, we could not adequately assess this relationship due to the variety of IRAR presentations.

We observed that 28% of IRAR presented with hemothorax and most (74%) were right sided, followed by bilateral hemothorax presentation (Table 2). Median sternotomy was the most commonly used surgical approach (62%) and was observed more when IRAR presented with a right hemothorax.

**Table 2:** Summary of all cases of isolated right atrial rupture from blunt trauma

Cardiac tamponade	Incidence	Mortality	Hospital LOS
Not present	35% (26)	19% (5) *	15 (0–99) *
Present	65% (49)	6% (3) *	9 (0–29) *
RAA	85% (22) *	9% (2) *	10 (0–27) *
RA/SVC	40% (8) *	13% (1)	8 (0–9) *
RA/IVC	44% (8) *	25% (2)	0 (0–20) *
Free RA wall	50% (6) *	0% (0)	17 (6–28) *
Concomitant hemothorax	16% (8)	13% (1)	10 (0–27)
Hemothorax	Incidence	Mortality	Hospital LOS
Not present	72% (54)	13% (7)	8 (0–82)
Present	17% (21)	14% (3)	15 (0–99)
RAA	15% (4)	25% (1)	10 (0–27)
RA/SVC	23% (3)	33% (1)	33 (7–79)
RA/IVC	42% (5)	0% (0)	14 (0–69)
Free RA wall	31% (5)	0% (0)	33 (7–99)
Surgical approaches	Median Sternotomy	R. Thoracotomy	L. Thoracotomy
All hemothorax	62% (13)	31.2% (5)	6.25% (3)
Right-sided	62.5% (10)	31.2% (5)	6.25% (1)
Left-sided	0% (0)	0% (0)	100% (1)
Bilateral	75% (3)	0%(0)	25% (1)

Median (range); proportion % (n); RA, right atrium; RAA, right atrial appendage; SVC/IVC, superior/inferior vena cava; LOS, length of stay. *Statistically significant difference (*p* < 0.05)

## Discussion

After reporting the first successful repair of a ruptured myocardium by in 1955, Desforges et al. believed that all chambers were equally susceptible to BCR [47]. Unfortunately, this theory was based on limited knowledge of BCRs from reports of sport-related sudden death during baseball games [47]. More recent autopsy studies have challenged this belief after observing that the right heart seemed to be more frequently ruptured [11]. However, autopsy studies do not provide individual patient data that specifically delineates whether or not each ruptured chamber was isolated. Thus, the exact incidence of IRAR is currently unclear and difficult to empirically assess. This study synthesizes a 62-year review of a rare condition with a unique etiology and critically challenging clinical features that may be applied to various settings.

The paucity in the relevant literature could primarily be attributed to IRAR’s high on-scene fatality [4].The vast majority of cases reported a MVA as the source of blunt trauma (Table 1). Other etiologies include, but are not limited to, falls, sports, weight lifting, combat, domestic violence, and more [10]. Of interest, Lu et al. reported a case of a new-born baby that suffered from IRAR due to the high-pressured descent through the vaginal wall during labour, demonstrating the variety of IRAR etiologies [15].

There are no clear risk factors for the IRAR. Although an acute myocardial infarction may increase the risk of cardiac rupture, the incidence of rupture in this population is less than 1%, which makes it very difficult to identify an association [48]. Furthermore, rupture from an infarcted cardiac wall will more likely involve the left ventricular or the interventricular septum, but not the RA [48]. Lastly, we observed that IRAR more commonly affect younger males, although seen in all ages, suggesting a weak correlation between IRAR and history of previous cardiac disease.

**Fig. 3:**
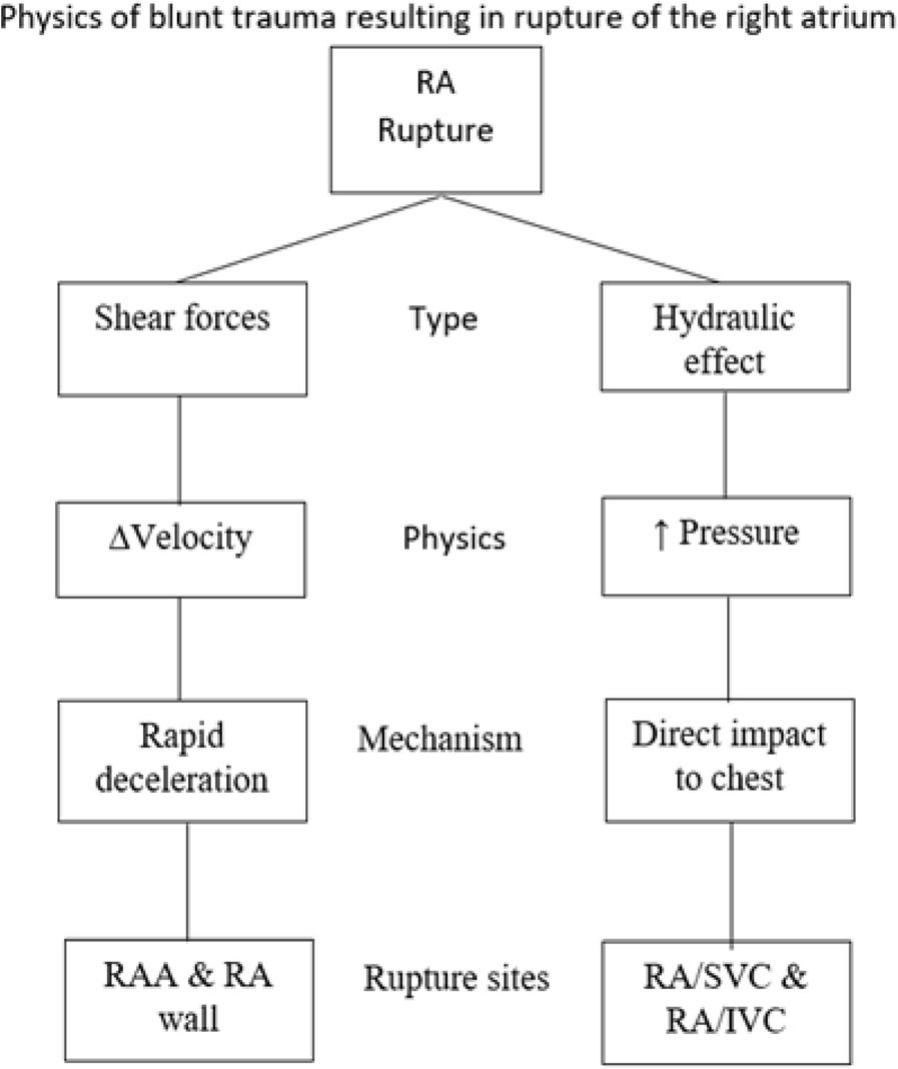
Flowchart illustrating the physics of right atrium rupture after blunt trauma

The mechanisms of blunt trauma resulting in IRAR are extensively detailed elsewhere [49, 50] (Fig. 3). Though complex, a brief understanding can elucidate more on its etiological heterogeneity. A theory set forth is the “hydraulic effect,” which was first proposed by Leavitt et al. as a mechanism of BCR in 1987 [4]. In summary, the hydraulic effect is a phenomenon observed when an applied force compresses a closed system, thereby decreasing its volume, is accompanied with an increase in pressure. The RA experiences the hydraulic effect on a daily basis during the cardiac cycle, particularly when the atrioventricular valve is closed in late systole [3–5]. Sudden increases in venous return can also increase the cardiac intraluminal pressure, which can be observed during normal inspiration, with abdominal compression, or with leg elevation [49]. Once the intraluminal pressure surpasses the cardiac wall elasticity, BCR occurs at the heart’s weakest point, the RA [49, 50].

Another mechanism of BCR that is more unique to IRAR is deceleration forces [50]. When a traveling vehicle abruptly changes direction or stops, the non-fixed, mobile heart will continue to travel in the same initial direction. Once the shearing forces surpass the cardiac elasticity, the RA may tear off the fixed superior vena cava (SVC) and IVC. Shearing forces have been more attributed to RA ruptures involving the fixed venal caval attachment sites, compared to the other two attachment sites [49, 50]. The RAA is the most common site of IRAR and this observation is mainly attributed to its thin wall (Table 1). The RAA’s wall is half as thick (0.53 ± 0.33 mm) comparted to the RA wall (1.11 ± 0.42 mm), making it more susceptible BCR [24].

The diagnosis is one of suspicion and continued hemodynamic instability often prompts the search for a bleeding source. However, many patients with IRAR are initially hemodynamically stable, which has been attributed to the RA’s lower intraluminal pressures. This “static” blood flow creates a more favorable environment for platelet aggregation and may sometimes form an intracardiac hematoma, can mimic the function of a bandage on the rupture site and may be beneficial by transiently decreasing blood loss and may relay a survival benefit [21].

The clinical presentations of BCRs are heterogeneous and vary depending on multiple factors including location and size [12]. Cardiac tamponade is a common feature of BCR and its presence may be an important prognostic factor in IRAR and may have a protective effect on mortality, which was statistically significant [9]. We observed a 6% mortality rate in patients with IRAR if they presented with cardiac tamponade (Table 2). This may be due to increased suspicion of a cardiac etiology when presented with cardiac tamponade, compared to potential delays in diagnosis and time to the operating room, associated with poorer outcomes, in patients that did not present with cardiac tamponade [46]. However, the clinical findings of “Beck’s triad” do not always present together, and up to 35% of cases do not present with cardiac tamponade (Table 1) [17]. Our patient developed profound hypotension but had weak carotid pulses with no jugular venous distension and auscultation of the heart was clear. However, the patient’s rapid hemodynamic decline did not provide enough time for adjunctive testing. Furthermore, there were no findings on FAST suggestive of cardiac tamponade making the diagnosis of rupture more difficult.

Hemothorax is not an uncommon presentation of BCR and is more likely as a result of a concomitant pericardial tear allowing extravasation of fluid into the thoracic cavity [7]. A right hemothorax is more likely with IRAR and the a left hemothorax is almost never present without involvement of the right side. Additionally, hemothorax is a common indication prompting a unilateral thoracotomy. However, median sternotomy is the best method to visualize the RA region as it allows for full visualization of the chest and is better tolerated [46]. Furthermore, up to 82% of blunt cardiac ruptures have associated abdominal injuries, and a median sternotomy may be easily extended into a laparotomy incision for further exploration of the abdomen [5]. We found in our review that most patients (62%) required median sternotomy (Table 2) Moreover, a pericardial window can be performed as a confirmatory test prior to a thoracotomy or a sternotomy and when other tests are inconclusive.

The RA has unique electrophysiological components and more than 80% of blunt cardiac injuries (BCIs) result in dysrhythmias [15]. It is unclear how BCIs causes dysrhythmias, but dysfunction of the vagal sympathetic innervating RA myocytes has been theorized, as well as disruption of the neuromuscular junctions between myocytes [56–58]. Furthermore, the type of dysrhythmia may help differentiated between atrial and ventricular injuries. This is because injury to the ventricles is more likely to produce a ventricular tachyarrhythmia or fibrillation. Conversely, RA injury is more likely to develop a bradyarrhythmia due to SA node dysfunction, similar to our patient that presented with profound bradycardia [15, 55].

Electrocardiogram (ECG) lacks specificity in the diagnosis of BCRs, as up to 58% of BCIs result in non-specific ECG changes [51–55]. Other tests, such as cardiac biomarkers (CBMs), have been proposed for the diagnosis of BCRs. However, CBMs also have been shown to lack specificity, as Mahmood et al. observed elevated CBMs in 20% of blunt chest trauma, even in the absence of cardiac involvement [60]. Recently, several studies, including a meta-analysis [54], have suggested the combined use of ECG and CBMs to rule out BCIs and/or monitor its progression [15]. Salim et al. conducted a prospective study of 115 patients with blunt chest trauma and found that both a normal ECG and non-elevation CBMs rules out most cases of BCIs [59]. Furthermore, they suggested that patients with abnormal ECG or CBMs may be monitored for 24–48-h and, with serial CBMs and ECG, be safely discharged in the absence of other injuries [59]. Ultimately, future studies will further elucidate the best role of CBMs in this condition and others.

## Conclusion

Based on our review, the isolated right atrial rupture from blunt cardiac trauma is most commonly from blunt traumas during motor vehicle accidents. There are no clear risk factors and the condition most commonly found in younger males. Patients may be initially hemodynamically stable due to smaller tears or hematomas, but clinicians should be cautious with consistent monitoring. Immediate operative intervention should be implemented for hemodynamic instability and short-term outcomes, with favourable outcomes if the condition is promptly managed. The presence of cardiac tamponade may have a protective effect, by increasing suspicion and prompting an investigation for a source of cardiac bleeding. In contrast, hemothorax and other concomitant injuries may worsen outcomes and have a poorer prognosis. The right atrial appendage is the most common location for rupture and may have better outcomes compared to other rupture sites. Further research is indicated as our knowledge of the condition increases.

## Abbreviations

BCI: Blunt cardiac injuries
BCR: Blunt cardiac rupture
CBMs: Cardiac biomarkers
FAST: Focused assessment with sonography for trauma
IPD: Individual Patient Data
IRAR: Isolated right atrial rupture
IVC: Inferior vena cava
LOS: Length of stay
MVA: Motor vehicle accident
RA: Right atrium
RAA: Right Atrial Appendage
SVC: Superior vena cava
